# MLD-Net: A Multi-Level Knowledge Distillation Network for Automatic Modulation Recognition

**DOI:** 10.3390/s25237143

**Published:** 2025-11-22

**Authors:** Xihui Zhang, Linrun Zhang, Meng Zhang, Zhenxi Zhang, Peiru Li, Xiaoran Shi, Feng Zhou

**Affiliations:** 1 Southwest China Institute of Electronic Technology, Chengdu 610036, China; seaharm_yeah@163.com (X.Z.); alice_mm@163.com (M.Z.); 2 Key Laboratory of Electronic Information Counter-Measure and Simulation, Xidian University, Xi’an 710071, China; zhanglinrun@stu.xidian.edu.cn (L.Z.); peiruli@stu.xidian.edu.cn (P.L.); xrshi@xidian.edu.cn (X.S.); 3 The School of Aerospace Science and Technology, Xidian University, Xi’an 710071, China; fzhou@mail.xidian.edu.cn

**Keywords:** automatic modulation recognition, knowledge distillation, Reformer, deep learning for communications

## Abstract

Automatic Modulation Recognition (AMR) is a critical technology for intelligent wireless communication systems, but the deployment of high-performance deep learning models is often hindered by their substantial computational and memory requirements. To address this challenge, this paper proposes a multi-level knowledge distillation network, namely MLD-Net, for creating a lightweight and powerful AMR model. Our approach employs a large Transformer-based network as a teacher to guide the training of a compact and efficient Reformer-based student model. The knowledge contained in the large model is transferred across three distinct granularities: at the output level, to convey high-level predictive distributions; at the feature level, to align intermediate representations; and at the attention level, to propagate relational information about signal characteristics. This comprehensive distillation strategy empowers the student model to effectively emulate the teacher’s complex reasoning processes. Experimental results on the RML2016.10A benchmark dataset demonstrate that MLD-Net achieves state-of-the-art performance, outperforming other baseline models across a wide range of signal-to-noise ratios while requiring only a fraction of the parameters. Extensive ablation study further confirms the collaborative contribution of each distillation level, validating that the proposed MLD-Net is an effective solution for developing lightweight and efficient AMR networks for edge deployment.

## 1. Introduction

With the explosive growth of wireless devices and the increasing scarcity of spectrum resources, efficiently managing and optimizing limited wireless resources has become a critical challenge [1]. Automatic Modulation Recognition (AMR), as a core technology in wireless communication systems, aims to identify the modulation scheme of received signals under unknown channel conditions. AMR is a key component for applications such as spectrum monitoring, interference detection, and cognitive radio [2].

However, traditional likelihood-based and feature-based AMR methods suffer from high computational complexity, strong reliance on prior knowledge, and limited feature representation capability, making it difficult to meet the dual demands of high accuracy and low latency in real-world scenarios [3].

In recent years, the rapid development of deep learning has brought significant breakthroughs to AMR. Large-scale deep neural networks can automatically extract highly discriminative features from raw signals in an end-to-end manner, significantly improving modulation recognition performance under complex channels and low signal-to-noise ratio (SNR) conditions [4,5]. However, these large models usually come with substantial computational and storage costs, which severely limit their real-time deployment on resource-constrained devices.

**Figure 1:**
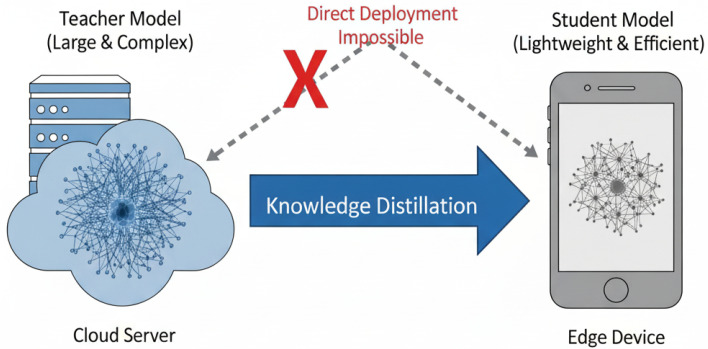
Illustration of knowledge distillation: a large teacher model transfers knowledge to a lightweight student, overcoming the limitations of direct deployment on edge devices for AMR.

Therefore, how to significantly reduce the computational and memory footprint of AMR models while maintaining high recognition accuracy has become a pressing research problem. To address this issue, Knowledge Distillation (KD) has emerged as an effective model compression technique. As illustrated in Figure 1, KD facilitates the transfer of knowledge from a large, high-performance “teacher” model (in our work, a standard Transformer encoder) to a lightweight “student” model (a compact Reformer). We specifically chose the Reformer architecture for its efficiency, as its core LSH (Locality-Sensitive Hashing) attention mechanism scales efficiently with sequence length (O(LlogL) complexity instead of $O(L^{2})$), making it ideal for resource-constrained edge deployment. This enables the compact student model to achieve strong model performance with much lower complexity, making it suitable for deployment on edge devices.

For AMR tasks, KD not only allows the student model to inherit the teacher’s ability to extract complex modulation features and global temporal–spectral dependencies but also improves the robustness of the student under noisy and dynamic channel conditions. Building upon the above background, this paper investigates the application of knowledge distillation to AMR. We present a knowledge distillation framework specifically designed for modulation recognition tasks, enabling efficient and robust performance under resource-constrained scenarios.

The main contributions of this study are summarized as follows:

- We propose a multi-level knowledge distillation network (MLD-Net) for AMR. By transferring knowledge simultaneously at the output, feature, and attention levels, MLD-Net enables a lightweight student model to learn not only the teacher’s predictive distribution but also its internal reasoning process regarding complex signal characteristics, significantly enhancing the student model’s performance and its robustness under diverse channel conditions.
- We design an efficient yet powerful student model. We demonstrate that a compact Reformer-based architecture, when guided by a larger Transformer teacher through our multi-level distillation, can achieve state-of-the-art accuracy with only a fraction of the computational and memory costs, making it ideal for edge deployment.
- Adequate comparative and ablation experiments on the RML2016.10A and RML2016.10B datasets validate that our proposed MLD-Net establishes a superior trade-off between efficiency and performance. It achieves state-of-the-art accuracy among lightweight models (e.g., 61.14% on 10A and 64.62% on 10B) with an approximately 131.9-fold parameter reduction compared to the teacher model.

The remainder of this paper is organized as follows. Section 2 reviews related work in automatic modulation recognition and model compression techniques. Section 3 details our proposed MLD-Net, including the signal model, the teacher-student architecture, and the multi-level distillation strategy. Section 4 presents the experimental setup, results, and ablation studies. Finally, Section 5 concludes the paper and discusses future work.

## 2. Related Work

### 2.1. Automatic Modulation Recognition

Traditional AMR methods can be roughly categorized into two classes: likelihood-based (LB) and feature-based (FB) approaches [6,7]. LB methods formulate AMR as a hypothesis testing problem, requiring prior knowledge of channel parameters such as signal-to-noise ratio (SNR) and frequency offset [8,9]. Typical LB techniques include average likelihood ratio test, generalized likelihood ratio test, and hybrid likelihood ratio test, which achieve optimal classification in the Bayesian sense [6,7]. However, they are computationally intensive and depend heavily on accurate prior knowledge, making them impractical in dynamic real-world environments [3].

In contrast, FB methods extract domain-specific features from signals, which are then fed into machine learning classifiers [10,11]. Classical examples include utilizing statistical features like high-order cumulants or cyclic spectral features, often classified by support vector machines [12] or random forests [13]. In recent years, more advanced FB approaches have shown promising results. For instance, methods based on phase diagrams and their entropy have been used for robust recognition [14,15], while compressive sensing techniques have been applied to achieve classification from sub-Nyquist samples [16,17]. However, the performance of these FB methods, both classical and modern, still relies heavily on the quality of handcrafted features and expert knowledge, which may struggle in complex and dynamic channel environments [3].

Recently, deep learning (DL) has shown strong feature learning capability in fields such as computer vision [18] and wireless communications [1]. DL-based AMR frameworks can learn discriminative features directly from raw signals without handcrafted feature engineering [5]. For example, initial works by O’Shea et al. [4] applied deep neural networks to raw IQ samples, while Peng et al. [19] used AlexNet and GoogLeNet on constellation diagrams. Deeper architectures, such as residual networks (ResNet), were also designed to extract more intricate features [20].

To better capture temporal and spatial relationships, subsequent research explored hybrid architectures. Prominent examples that combine CNNs for feature extraction and LSTMs for temporal modeling include the widely used CNN+LSTM [21] and the deeper ResNet+LSTM [22]. Other powerful models include CNNIQ, a network designed for robustness under fading channels [23], and CLDNN, which integrates CNN, LSTM, and fully connected layers [24]. An extension, MCLDNN, further enhances performance through a spatiotemporal multi-channel learning framework [25]. More recently, Transformer-based architectures have been introduced to model long-range dependencies in signals effectively [26,27,28], setting a new benchmark for performance.

### 2.2. Model Pruning and Lightweight

With the growing adoption of deep learning in communication and other domains, the computational and memory overhead of large models has become a major bottleneck for real-time inference and deployment on edge devices. This is particularly critical in AMR tasks, which require fast and efficient models.

To address this, various lightweight techniques have been developed. For instance, quantization [29] reduces model size by lowering the numerical precision of weights and activations, with quantization-aware training (QAT) often mitigating the performance degradation associated with post-training quantization (PTQ). Another common method is pruning [30], which is based on the “lottery ticket hypothesis” and removes non-essential parameters. Pruning can be unstructured, by sparsifying weights, or structured, by removing entire components like channels or attention heads to achieve acceleration on general hardware.

Beyond adjusting parameters, architectural modifications also play a key role. Knowledge distillation transfers knowledge from a large teacher model to a compact student, enabling the student to learn rich representations, as demonstrated by TinyBERT [31,32]. Parameter sharing, as implemented in ALBERT, reuses weights across layers to achieve significant model compression [33].

Furthermore, to address the quadratic complexity of self-attention on long sequences, efficient attention mechanisms have been proposed. Reformer [34] leverages locality-sensitive hashing (LSH) to reduce complexity to O(LlogL) and uses reversible residual layers to save memory. Similarly, Linformer [35] approximates the attention matrix via low-rank projection, reducing complexity to O(L). Overall, these techniques can be combined to compress model size, accelerate inference, and reduce energy consumption, providing practical solutions for deploying deep models in resource-constrained AMR scenarios.

### 2.3. Knowledge Distillation

Knowledge distillation (KD) has emerged as an effective technique for compressing and accelerating deep models by transferring knowledge from a large teacher model to a lightweight student model. In AMR tasks, KD enables student models to retain high recognition accuracy while meeting the computational and memory constraints of edge devices.

The concept of KD was first introduced by Hinton et al. [32], who proposed that students should learn from the soft probability distribution output by the teacher, which encodes inter-class similarity and enhances generalization. Later, FitNets [36] extended KD by guiding the student to also learn intermediate feature representations from the teacher, improving performance further. Zagoruyko and Komodakis [37] proposed attention transfer, encouraging the student to focus on the same salient regions as the teacher.

To narrow the gap between teacher and student, more advanced strategies have been developed. Furlanello et al. [38] explored multi-generation distillation, where students iteratively teach subsequent students. Kimura et al. [39] introduced pseudo-samples to strengthen student training in few-shot scenarios. Park et al. [40] proposed a relational knowledge distillation approach, constructing distance-wise and angle-wise distillation losses to transfer the interrelationships of instances from the teacher to the student. Liu et al. [41] designed a method to preserve the inter-channel correlation of features during distillation.

In AMR, the modulation patterns are often buried in complex temporal, spectral, and amplitude variations, and subject to noise and interference. KD allows the student model to benefit from the teacher’s ability to extract robust and discriminative signal features. Combining KD with feature selection, multi-scale modeling, and attention mechanisms further enhances student performance under challenging conditions, making KD an indispensable approach for efficient AMR model design.

## 3. Methodology

In this section, we present our proposed lightweight automatic modulation recognition (AMR) framework based on multi-level knowledge distillation. We detail the signal model, the proposed teacher-student architecture, and the multi-level distillation strategy used for training.

### 3.1. Signal Model

In a typical wireless communication system, the transmitted signal is subjected to various channel impairments. To accurately model this process, we consider a complex baseband representation of the received signal. The channel model incorporates key distortions simulated in platforms like GNU Radio, such as multipath fading, carrier frequency offset (CFO), phase offset (PO), and additive white Gaussian noise (AWGN).

The received signal r(t) at the input of the receiver can be expressed as:

(1) $$r\left(t\right)=\left(s(t)*h(t)\right)e^{j(2\pi \Delta ft+\phi )}+n\left(t\right)$$

where s(t) is the complex baseband transmitted signal, the operator ∗ denotes convolution, and h(t) is the channel impulse response modeling the multipath effects. The term $e^{j(2\pi \Delta ft+\phi )}$ represents the impact of oscillator inaccuracies, with Δf being the carrier frequency offset and ϕ the phase offset. Finally, n(t) is the complex additive white Gaussian noise. This signal is then sampled to obtain a sequence of in-phase (I) and quadrature (Q) components, which is formatted as a 2×L matrix for model input, where the sequence length L=128.

### 3.2. Proposed Framework Architecture

**Figure 2:**
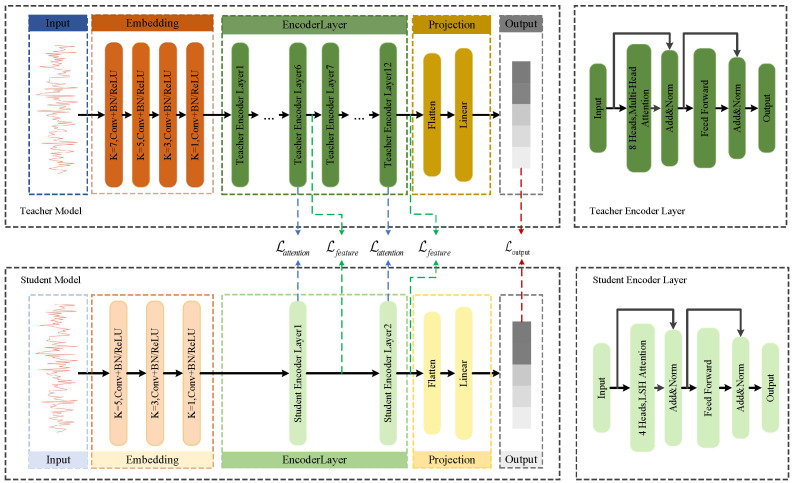
The proposed multi-level knowledge distillation framework. It illustrates the teacher-student architecture with highlighted knowledge distillation paths (1) $L_{\mathrm{attention}}$, (2) $L_{\mathrm{feature}}$, and (3) $L_{\mathrm{output}}$. Detailed structures of the Teacher (Transformer) and Student (Reformer) Encoder Layers are provided on the right.

Our proposed framework is built upon a teacher-student paradigm, as illustrated in Figure 2. The core idea is to transfer knowledge from a large, high-performance teacher model to a compact and efficient student model, which is based on the Reformer architecture. The teacher model is a standard Transformer encoder designed for maximum accuracy, while the student model is an efficient Reformer designed for lightweight deployment.

A key differentiator between the teacher and student models lies in their attention mechanisms. The teacher model employs standard Full Multi-Head Self-Attention, which computes attention scores between every pair of tokens in the input sequence. While powerful, this approach has a computational and memory complexity of $O(L^{2})$ with respect to the sequence length *L*, making it demanding for long sequences.

In contrast, the student model utilizes LSH (Locality-Sensitive Hashing) Attention, a core innovation of the Reformer architecture. Instead of performing a full comparison, LSH Attention uses hashing to group similar tokens together and computes attention only within these smaller, localized groups. This strategy significantly reduces the complexity to a much more manageable O(LlogL), enabling efficient processing of longer sequences with a smaller memory footprint.

To be specific, the student model is a 2-layer Reformer encoder, as detailed in Table 1 and visually depicted in Figure 2. It utilizes LSH Attention with 4 attention heads, 4 hashes, and a bucket size of 32 to approximate the full attention mechanism. The model dimension ($d_{\mathrm{model}}$) is reduced to 128, and it does not use reversible residual layers to optimize for inference speed. This lightweight design is the key to its efficiency.

The detailed architectural comparison, including layer counts, model dimensions, and attention types, is presented in Table 1. As shown, the student model achieves an approximately 131.9-fold reduction in parameters (from 38.27 M to 0.29 M), highlighting its suitability for resource-constrained environments. The intricate connections and distillation points are further illustrated in Figure 2.

### 3.3. Multi-Level Knowledge Distillation

To endow the compact Reformer student with the capabilities of the powerful teacher model, we employ a multi-level knowledge distillation strategy. This strategy transfers knowledge at three distinct granularities, compelling the student to mimic the teacher’s behavior from low-level feature extraction to high-level decision-making. The student model is subsequently trained by minimizing a composite loss function that synergizes a standard supervised loss with these multi-level distillation losses.

The first level is output-level distillation, which transfers the teacher’s class probability distribution to the student. This is achieved by minimizing the Kullback-Leibler (KL) divergence between the softened logits of the two models. Let $p_{t}=\sigma \left(z_{t}/T\right)$ and $p_{s}=\sigma \left(z_{s}/T\right)$ be the temperature-scaled probability distributions from the teacher and student, respectively, where σ(·) is the softmax function. The loss is defined as:

(2) $$L_{\mathrm{output}}=T^{2}\cdot D_{\mathrm{KL}}(p_{t}\left|\right|p_{s})=T^{2}\sum_{i=1}^{C}p_{t}^{\left(i\right)}\log\left(\frac{p_{t}^{\left(i\right)}}{p_{s}^{\left(i\right)}}\right).$$

where $z_{s}$ and $z_{t}$ are the logit vectors, *C* is the number of modulation classes, and *T* is a temperature hyperparameter. Following the original knowledge distillation framework [32], we multiply the KL divergence loss by a factor of $T^{2}$. This scaling is crucial because the gradients produced by the soft targets scale as $1/T^{2}$. Including the $T^{2}$ term ensures that the relative contribution of the distillation loss and the standard cross-entropy loss remains roughly constant when the temperature is changed.

The second level, feature-level distillation, transfers the rich intermediate representations learned within the teacher’s hidden layers. To align these representations, we define a formal mapping between specific layers of the student and teacher models. Let M be the set of index pairs (i,j), where the *i*-th layer of the student model corresponds to the *j*-th layer of the teacher model. In our framework, which maps a 12-layer teacher to a 2-layer student, this set is defined as M={(1,6),(2,12)}. This specific mapping was chosen to align the student’s layers with proportionally deep layers in the teacher. The student’s first layer (layer 1 of 2) is mapped to the teacher’s middle layer (layer 6 of 12) to learn robust, mid-level representations. The student’s final layer (layer 2 of 2) is mapped to the teacher’s final layer (layer 12 of 12) to capture the most abstract, high-level features and attention patterns just before the final projection. The feature distillation loss is then defined as the mean squared error between the corresponding feature maps, averaged over all mapped pairs and all samples in a batch:

(3) $$L_{\mathrm{feature}}=\frac{1}{N|M|}\sum_{n=1}^{N}\sum_{(i,j)\in M}{∥W_{p}\left(f_{s,n}^{\left(i\right)}\right)-f_{t,n}^{\left(j\right)}∥}_{2}^{2}.$$

where $f_{s,n}^{\left(i\right)}$ is the feature representation for the *n*-th sample from the *i*-th layer of the student, and $f_{t,n}^{\left(j\right)}$ is from the *j*-th layer of the teacher. *N* is the number of samples in the batch, and |M| is the number of mapped layer pairs. As the feature dimensionalities may differ, a trainable linear projection layer, $W_{p}$, is employed to match the student’s feature space to the teacher’s.

The third level is attention-level distillation, which transfers the relational knowledge encoded in the self-attention mechanisms. This relational knowledge is distinct from and complementary to the feature-level distillation. The feature-level loss, $L_{\mathrm{feature}}$, is designed to align the student’s intermediate representations with the teacher’s. These representations typically provide a holistic summary of the signal’s learned characteristics, often pooled or summarized, teaching the student what high-level features to extract, regardless of their specific temporal positions. In contrast, the attention loss $L_{\mathrm{attention}}$ operates directly on the full L×L self-attention matrices. These matrices explicitly model the temporal dependencies and token-to-token relationships within the sequence. This forces the student to mimic how the teacher model weighs the relative importance of different time steps in the I/Q sequence. For AMR, this distinction is crucial: $L_{\mathrm{feature}}$ ensures the student learns a robust set of features, while $L_{\mathrm{attention}}$ transfers the teacher’s complex temporal reasoning about which parts of the signal are most salient for classification. This is achieved by minimizing the KL divergence between the student’s and teacher’s attention distributions. The loss is calculated as the average over all samples in a batch and all mapped layer pairs. The loss function is defined as:

(4) $$L_{\mathrm{attention}}=\frac{1}{N|M|}\sum_{n=1}^{N}\sum_{(i,j)\in M}\left(D_{\mathrm{KL}}(P_{t,n}^{\left(j\right)}\left|\right|P_{s,n}^{\left(i\right)})+\lambda H\left(P_{t,n}^{\left(j\right)}\right)\right).$$

where $P_{s,n}^{\left(i\right)}$ and $P_{t,n}^{\left(j\right)}$ are the attention distributions for the *n*-th sample in the corresponding mapped layers. $D_{\mathrm{KL}}\left(\cdot ||\cdot \right)$ denotes the KL divergence, and $H\left(P\right)=-\sum_{k}P_{k}\log P_{k}$ is the Shannon entropy. The term λ is a hyperparameter that weights the entropy regularization on the teacher’s attention distribution, which encourages a smoother learning signal.

The training objective is also guided by the standard supervised learning loss, which is the cross-entropy loss ($L_{\mathrm{cls}}$) between the student model’s predictions and the ground-truth labels. This loss is defined as:

(5) $$L_{\mathrm{cls}}=-\frac{1}{N}\sum_{n=1}^{N}\sum_{c=1}^{C}y_{n,c}\log\left(p_{s,n,c}\right).$$

where $y_{n,c}$ is the one-hot encoded ground-truth label for the *n*-th sample and *c*-th class, and $p_{s,n,c}$ is the student’s predicted probability.

The final composite loss function is a weighted sum of the standard cross-entropy loss and the three aforementioned distillation losses:

(6) $$L_{\mathrm{total}}=\alpha L_{\mathrm{cls}}+\beta L_{\mathrm{output}}+\gamma L_{\mathrm{feature}}+\delta L_{\mathrm{attention}}.$$

where α,β,γ,δ are scalar hyperparameters that balance the contribution of each loss component. In our experiments, these were empirically set to α=0.5, β=0.5, γ=0.3, and δ=0.3. This comprehensive loss function guides the student model to not only learn the correct classifications but also to internalize the intricate knowledge and reasoning process of the larger teacher model.

## 4. Experiments

### 4.1. Dataset and Experimental Setup

This study employs two publicly available datasets, RML2016.10A and RML2016.10B, as standard benchmarks for AMR research. Both datasets contain signals synthetically generated using the GNU Radio platform to emulate a real-world wireless communication environment, incorporating various channel impairments such as AWGN, multipath fading, clock synchronization errors, and carrier frequency offsets. All samples comprise 128 I/Q data points, generated at a nominal 8 samples-per-symbol, and span SNRs from −20 dB to +18 dB. The RML2016.10A dataset contains 220,000 samples across 11 modulation types, while RML2016.10B provides 1.2 million samples across 10 modulation types (excluding AM-SSB). For our experiments, each dataset was partitioned into training, validation, and testing sets using a 6:2:2 ratio.

All models were implemented using PyTorch (version 1.13.1) and trained on a single NVIDIA GeForce RTX 3060 GPU. The training process for MLD-Net was two-staged. First, the teacher model was trained from scratch until convergence using the Adam optimizer with a learning rate of $1\times 10^{-4}$ and a weight decay of $1\times 10^{-5}$. Second, the student model was trained using the multi-level distillation framework with the pre-trained teacher model’s parameters frozen. All baseline models and the student model were trained using a learning rate of $1\times 10^{-3}$ and a weight decay of $1\times 10^{-4}$.

For all training, a batch size of 128 was used. In the distillation stage, the temperature *T* was set to 3.0, and the loss weights were set to α=0.5, β=0.5, γ=0.3, and δ=0.3. The primary evaluation metric is the overall classification accuracy across all SNRs.

### 4.2. Results

In this section, we evaluate the performance of our proposed method, which we name MLD-Net. We first compare MLD-Net with several state-of-the-art AMR models in terms of both model efficiency and classification accuracy. Then, we conduct an ablation study to analyze the contribution of each component in our multi-level distillation framework.

#### 4.2.1. Comparison with State-of-the-Art Methods

We compare our proposed MLD-Net against its un-distilled version (Student w/o KD), the large teacher model, and seven baseline models: CLDNN [24], CNN+LSTM [21], CNNIQ [23], MCLDNN [25], ResNet+LSTM [22], MobileNet [42], and ShuffleNet [43].

To provide a comprehensive evaluation, Table 2 summarizes the model complexity, including the number of parameters, FLOPs, and inference time, alongside the overall classification accuracy on both RML2016.10A and RML2016.10B datasets.

The results clearly demonstrate the effectiveness of our knowledge distillation framework. The large teacher model, while achieving the highest accuracy, is computationally prohibitive in terms of both parameters (38.27 M) and computational cost (3.263 G FLOPs). Through our MLD-Net framework, the student model significantly improves its performance over the baseline: on RML2016.10A, accuracy increases from 59.06% to 61.14%, and on RML2016.10B, it increases from 61.80% to 64.62%.

This performance is achieved while maintaining an approximately 131.9-fold reduction in parameters compared to the teacher.

Furthermore, MLD-Net establishes a new state-of-the-art trade-off between efficiency and performance. A detailed analysis of Table 2 reveals the critical relationship between computational cost and model accuracy. In terms of FLOPs, our MLD-Net requires 35.899 M, which is a moderate cost. While ultra-lightweight models like ShuffleNet and MobileNet offer significantly lower FLOPs at 0.873 M and 2.812 M respectively, this extreme compression results in a clear performance deficit, as evidenced by their lower overall accuracy. This trade-off is also evident in the Inference Time. Models such as CNNIQ and CNN+LSTM achieve the fastest speeds, clocking in as low as 0.4754 ms. However, this speed comes at the cost of accuracy, with their performance lagging behind our model. Our MLD-Net, with inference times of 1.5674 ms on RML2016.10A and 1.1495 ms on RML2016.10B, makes a deliberate trade-off. It leverages a moderate computational and inference budget to achieve the highest accuracy among all non-teacher models on both datasets. Specifically, MLD-Net achieves 61.14% on dataset 10A and 64.62% on dataset 10B, nearly matching the performance of the full-sized teacher model. This highlights our model’s ability to establish a superior balance between a practical, real-time inference speed and state-of-the-art accuracy, making it highly suitable for deployment on edge devices.

**Figure 3:**
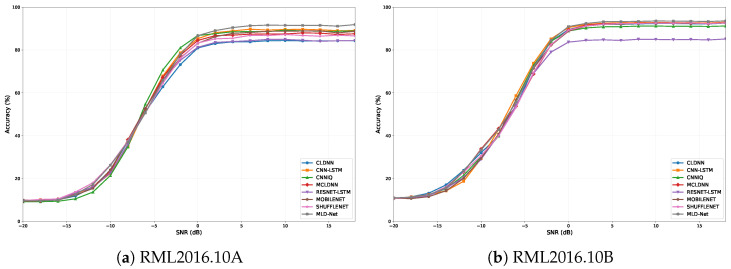
Classification accuracy versus SNR for the proposed MLD-Net and baseline models on (**a**) RML2016.10A and (**b**) RML2016.10B.

The superior classification performance of MLD-Net is further detailed in Figure 3, which illustrates the accuracy of all models across the full range of SNRs from −20 dB to +18 dB on both the RML2016.10A and RML2016.10B datasets. It is evident that MLD-Net consistently outperforms all baseline models, particularly in the mid-to-high SNR region (0 dB to 18 dB) on both datasets. While all models exhibit similar performance at very low SNRs where noise dominates, our model’s accuracy curve rises more steeply and achieves a higher plateau, demonstrating its superior robustness and feature extraction capabilities inherited from the teacher model.

**Figure 4:**
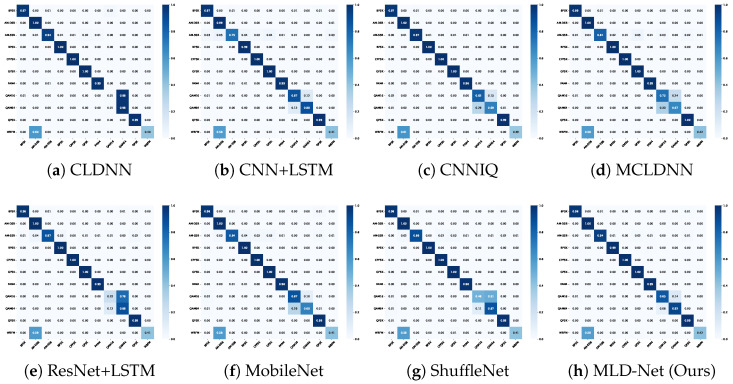
Confusion matrices of different models at SNR = 10 dB on the RML2016.10A dataset.

To further analyze the classification performance, we present the confusion matrices for all models at an SNR of 10 dB on the RML2016.10A dataset in Figure 4. We selected 10 dB as it is a representative mid-to-high SNR where model differences are clearly visible. This point offers the most informative comparison of how each model handles challenging, spectrally similar classes, such as QAM16 and QAM64, once performance has stabilized from the low-SNR regime. A darker diagonal indicates higher accuracy for individual modulation types. The confusion matrix for our MLD-Net (h) shows a significantly more pronounced diagonal compared to the baselines. Notably, baseline models like CLDNN (a) and CNN+LSTM (b) exhibit considerable confusion between spectrally similar modulations, such as QAM16 and QAM64, or AM-SSB and WBFM. In contrast, our model demonstrates a marked improvement in discriminating these challenging classes, which validates the effectiveness of the transferred knowledge.

**Figure 5:**
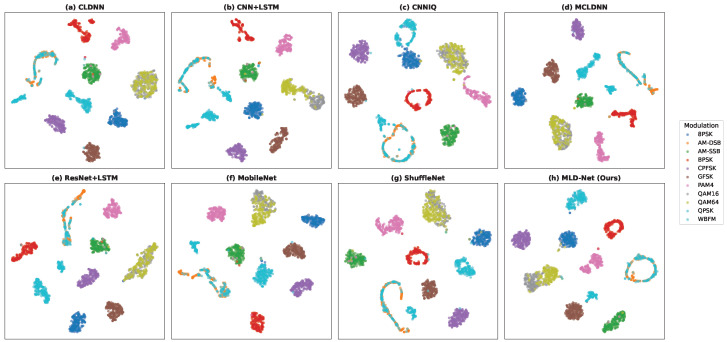
t-SNE visualization of feature distributions from different models at SNR = 10 dB on the RML2016.10A dataset.

Finally, to visualize the learned feature representations, we use the t-SNE technique to project the high-dimensional features from the models’ final hidden layer into a 2D space. As shown in Figure 5 (for the RML2016.10A dataset), the feature clusters produced by MLD-Net (h) are significantly more compact and well-separated compared to the baseline models. This visual evidence confirms that our multi-level distillation approach enables the lightweight student model to learn highly discriminative features, leading to improved classification performance.

#### 4.2.2. Ablation Study

To validate the effectiveness and complementarity of each component within our proposed MLD-Net, we conduct a comprehensive ablation study, as requested by Reviewer 1. We compare the performance of our full model with several variants on both the RML2016.10A (A) and RML2016.10B (B) datasets. The experimental configurations are as follows:

- Student w/o KD: The baseline Reformer student model trained from scratch using only the cross-entropy loss ($L_{\mathrm{cls}}$).
- +Output KD: Student trained with $L_{\mathrm{cls}}+L_{\mathrm{output}}$.
- +Feature KD: Student trained with $L_{\mathrm{cls}}+L_{\mathrm{feature}}$.
- +Attention KD: Student trained with $L_{\mathrm{cls}}+L_{\mathrm{attention}}$.
- +Output + Feature: Student trained with the two corresponding distillation losses.
- +Output + Attention: Student trained with the two corresponding distillation losses.
- +Feature + Attention: Student trained with the two corresponding distillation losses.
- MLD-Net (Ours): The full proposed model trained with all four loss components.

The results of the ablation study are summarized in Table 3. The baseline student model (Student w/o KD) achieves a modest accuracy on both datasets. The addition of any single distillation component (Output, Feature, or Attention KD) brings a noticeable performance improvement, confirming their individual effectiveness.

More importantly, the results demonstrate a clear synergistic effect. Combining pairs of distillation methods (e.g., +Output + Feature) yields superior results compared to any single method alone. Our full MLD-Net, which leverages all three distillation losses simultaneously, achieves the highest accuracy on both datasets (61.14% on 10A and 64.62% on 10B). This validates our core hypothesis: the different knowledge types are complementary, and a multi-level approach that transfers output distributions, feature representations, and relational attention patterns is essential for maximizing the performance of the lightweight student model.

## 5. Conclusions

In this paper, we proposed MLD-Net, a novel multi-level knowledge distillation framework designed to produce a lightweight yet high-performance model for Automatic Modulation Recognition. By leveraging a large Transformer-based teacher model, we successfully transferred comprehensive knowledge to a compact Reformer-based student model. Our approach goes beyond traditional output-level distillation by incorporating feature-level and attention-level distillation, which compels the student to mimic the teacher’s internal feature representations and relational reasoning patterns. This multi-faceted knowledge transfer enables the student model to achieve significant performance gains that would be unattainable through standard training alone. Experimental results on the public RML2016.10A and RML2016.10b datasets demonstrate the superiority of our MLD-Net. It achieves state-of-the-art accuracy (61.14% on 10a and 64.62% on 10b) among lightweight models. With an approximately 131.9-fold parameter reduction, our model consistently outperforms several state-of-the-art AMR baselines across a wide range of SNRs. Our work provides a robust and effective solution for deploying advanced AMR capabilities on resource-constrained edge devices.

We acknowledge, however, some limitations. Our positive results are demonstrated on the widely used RML synthetic datasets. While these datasets include channel impairments, real-world deployment on edge devices will present further challenges, such as hardware-specific nonlinearities and unseen co-channel interference. Nonetheless, the MLD-Net’s drastically reduced parameter count (0.29 M) and fast inference time (approx. 1.1–1.6 ms/sample) make it a strong and viable candidate for implementation in real-time systems, where these additional challenges can be addressed.

Future work could explore the application of MLD-Net to more complex and diverse signal datasets, as well as investigate the integration of other model compression techniques, such as quantization and pruning, to further optimize the model for real-world deployment.

## Figures and Tables

**Table 1:** Architectural comparison of the Teacher and Student models.

Component	Teacher Model	Student Model
Signal Embedding	Deep 4-layer CNN (2 channels → 512 channels)	Lightweight 3-layer CNN (2 channels → 128 channels)
Backbone Type	Standard Transformer Encoder	Reformer Encoder
Number of Layers	12	2
Model Dimension ($d_{\mathrm{model}}$)	512	128
Attention Type	Full Multi-Head Self-Attention	LSH Attention
Number of Heads	8	4
Total Parameters	38.27 M	0.29 M

**Table 2:** Comparison of model performance on two datasets (A: RML2016.10A, B: RML2016.10B).

Model	Dataset	Parameters	FLOPs	SNR (dB)		Overall	Inference Time (ms/Sample)
				−20–−2	0–18		
Teacher	A	38.27 M	3.263 G	31.77%	90.71%	61.24%	2.9612
	B			35.85%	93.46%	64.65%	2.9979
CLDNN	A	1.03 M	15.680 M	30.48%	83.73%	57.10%	0.7107
	B			36.63%	91.90%	64.26%	0.7061
CNN+LSTM	A	0.50 M	16.622 M	30.43%	88.74%	59.58%	0.6006
	B			35.84%	92.17%	64.01%	0.5658
CNNIQ	A	0.87 M	9.149 M	31.98%	88.23%	60.11%	0.4754
	B			35.86%	90.50%	63.18%	0.5604
MCLDNN	A	1.16 M	34.358 M	33.02%	86.82%	59.92%	1.0613
	B			36.62%	90.94%	63.78%	1.0430
ResNet+LSTM	A	7.40 M	35.032 M	32.74%	83.74%	58.24%	1.7717
	B			35.98%	84.58%	60.28%	1.8139
MobileNet	A	0.21 M	2.812 M	32.21%	86.89%	59.55%	1.3535
	B			35.04%	91.52%	63.28%	1.0255
ShuffleNet	A	0.06 M	0.873 M	31.79%	86.03%	58.91%	2.5073
	B			35.62%	91.26%	63.44%	2.6010
Student w/o KD	A	0.29 M	35.899 M	30.43%	87.68%	59.06%	1.5686
	B			32.97%	90.62%	61.80%	1.1479
MLD-Net (Ours)	A	0.29 M	35.899 M	31.62%	90.65%	61.14%	1.5674
	B			36.20%	93.03%	64.62%	1.1495

**Table 3:** Ablation study on RML2016.10A (A) and RML2016.10B (B) datasets.

Method	Dataset	$L_{\mathrm{cls}}$	$L_{\mathrm{output}}$	$L_{\mathrm{feature}}$	$L_{\mathrm{attention}}$	Accuracy
Student w/o KD	A	✓				59.06%
	B	✓				61.80%
+Output KD	A	✓	✓			60.21%
	B	✓	✓			63.15%
+Feature KD	A	✓		✓		59.88%
	B	✓		✓		63.02%
+Attention KD	A	✓			✓	59.76%
	B	✓			✓	62.85%
+Output + Feature	A	✓	✓	✓		60.65%
	B	✓	✓	✓		64.25%
+Output + Attention	A	✓	✓		✓	60.34%
	B	✓	✓		✓	63.79%
+Feature + Attention	A	✓		✓	✓	59.95%
	B	✓		✓	✓	63.45%
MLD-Net (Ours)	A	✓	✓	✓	✓	61.14%
	B	✓	✓	✓	✓	64.62%

## Data Availability

The RML2016.10A and RML2016.10B datasets presented in this study are publicly available from DeepSig Inc. at https://www.deepsig.ai/datasets (accessed on 19 November 2025).
